# Supplementary material for: A cross-sectional study examining generalized anxiety disorder among healthcare workers during the COVID-19 pandemic within the Democratic Republic of the Congo
Source: PLOS Ment Health. 2025 Aug 18;2(8):e0000224. doi: 10.1371/journal.pmen.0000224 (PMC12798435; doi:10.1371/journal.pmen.0000224)
Supplement: Attachment — Submitted filename: Anxiety_HCW_DRC_ReviewerResponses.docx [file pmen.0000224.s004.docx]

Response to Reviewers Manuscript ID: PMEN-D-24-00557 Title: A Cross-Sectional Study Examining Generalized Anxiety Disorder Among Healthcare Workers During the COVID-19 Pandemic Within the Democratic Republic of the Congo Corresponding Author: Dr. Anne W. Rimoin

Dear Editors and Reviewers,

We sincerely thank you for the thoughtful and constructive feedback on our manuscript. We are grateful to both reviewers for their time and expertise, and to the editorial team for the opportunity to revise and resubmit. We have carefully considered and addressed every comment, and we believe the manuscript is significantly improved as a result.

Below, we provide a point-by-point response to all editorial and reviewer comments. Reviewer comments are listed, and our responses follow each in blue text. We have indicated where revisions were made in the manuscript and cite line numbers based on the final clean version.

1. Please include a complete copy of PLOS’ questionnaire on inclusivity in global research in your revised manuscript. Our policy for research in this area aims to improve transparency in the reporting of research performed outside of researchers’ own country or community. The policy applies to researchers who have travelled to a different country to conduct research, research with Indigenous populations or their lands, and research on cultural artefacts. The questionnaire can also be requested at the journal’s discretion for any other submissions, even if these conditions are not met.  Please find more information on the policy and a link to download a blank copy of the questionnaire here: https://journals.plos.org/plosmentalhealth/s/best-practices-in-research-reporting. Please upload a completed version of your questionnaire as Supporting Information when you resubmit your manuscript.

Please see attached.

2. In the ethics statement in the Methods, you have specified that verbal consent was obtained. Please provide additional details regarding how this consent was documented and witnessed, and state whether this was approved by the IRB.

Per our updated manuscript: This assessment was deemed a public health surveillance activity by the Centers for Disease Control (CDC) and the University of California, Los Angeles (UCLA) and was granted a non-research determination; ethical approval was given in-country by the University of Kinshasa, Kinshasa School of Public Health Ethics Committee (IRB: ESP/CE/128/2021). Verbal consent procedures were reviewed and approved by the IRB and were documented by trained staff within the survey software.

3. We do not publish any copyright or trademark symbols that usually accompany proprietary names, eg (R), (C), or TM (e.g. next to drug or reagent names). Please remove all instances of trademark/copyright symbols throughout the text, including ® and ™ on page 17.

All copyright and trademark symbols have been removed.

Reviewers' comments:

Reviewer's Responses to Questions

**Comments to the Author**

1. Does this manuscript meet PLOS Mental Health’s [publication criteria](https://journals.plos.org/mentalhealth/s/criteria-for-publication)? Is the manuscript technically sound, and do the data support the conclusions? The manuscript must describe methodologically and ethically rigorous research with conclusions that are appropriately drawn based on the data presented.

Reviewer #1: Partly

Reviewer #2: Yes

2. Has the statistical analysis been performed appropriately and rigorously?

Reviewer #1: Yes

Reviewer #2: Yes

3. Have the authors made all data underlying the findings in their manuscript fully available (please refer to the Data Availability Statement at the start of the manuscript PDF file)?

The [PLOS Data policy](https://journals.plos.org/mentalhealth/s/data-availability) requires authors to make all data underlying the findings described in their manuscript fully available without restriction, with rare exception. The data should be provided as part of the manuscript or its supporting information, or deposited to a public repository. For example, in addition to summary statistics, the data points behind means, medians and variance measures should be available. If there are restrictions on publicly sharing data—e.g. participant privacy or use of data from a third party—those must be specified.

Reviewer #1: Yes

Reviewer #2: No

4. Is the manuscript presented in an intelligible fashion and written in standard English?

PLOS Mental Health does not copyedit accepted manuscripts, so the language in submitted articles must be clear, correct, and unambiguous. Any typographical or grammatical errors should be corrected at revision, so please note any specific errors here.

Reviewer #1: Yes

Reviewer #2: Yes

5. Review Comments to the Author

Please use the space provided to explain your answers to the questions above. You may also include additional comments for the author, including concerns about dual publication, research ethics, or publication ethics. (Please upload your review as an attachment if it exceeds 20,000 characters)

Reviewer #1: Manuscript ID: PMEN-D-24-00557–titled: “A Cross-Sectional Study Examining Anxiety Levels of Healthcare Workers and Community Members Within the Democratic Republic of the Congo”

I read with interest this manuscript, which is very interesting and presents original findings, to the best of my knowledge. First, I want to congratulate the authors on the quality of their work. This manuscript has the merit of being published soon if the authors consider certain aspects to improve the quality of its content.
Thank you for your kind comments and detailed feedback!

General remarks and advice
After careful reading of this manuscript, I understand that this study sought to estimate the prevalence of generalized anxiety disorders among healthcare workers (accounted for 77% of the sample) who were confronted with the Covid-19 pandemic, then to determine the anxiety levels in those who suffered from them and to identify the associated factors with the risk of developing these anxiety disorders. I understand that the general population was also included in the broad Covid vaccination study. However, including the general population (community members) in this highly relevant study seems to dilute its focus.

We appreciate the reviewer’s perspective. Inclusion of the general population was intentional and important for two key reasons. First, while HCWs were a key subgroup of interest, this study aimed to evaluate the broader mental health impact of the COVID-19 pandemic across diverse demographics within the DRC. Including both HCWs and community members enabled us to explore whether occupation modified risk and allowed for population-level insights. Second, our regression models required the full sample to assess the relative influence of factors such as housing, vaccination, and regional insecurity. Restricting the sample to HCWs alone would have limited statistical power and interpretability. We have clarified this rationale in the Methods and Discussion sections.

If the authors used the “7-item generalized anxiety disorder (GAD-7) scale” as a data collection tool, I suggest that, from the title right through to the body of the manuscript, they clearly refer to “generalized anxiety disorder” and not primarily to “anxiety level”.
It is also important to emphasize the key concept “COVID-19 pandemic” in the title. Unless the authors find it inappropriate, wording such as “A Cross-Sectional Study Examining Generalized Anxiety Disorders Among Healthcare Workers During The COVID-19 Pandemic Within the Democratic Republic of the Congo” may be clear to potential readers.

I would suggest that the authors do not place too much emphasis in this analysis on the association between “politically insecure regions and anxiety levels”. This aspect is more complex than it is easy to address in a cross-sectional study such as this.

We agree with your comment regarding the title and have updated it to reflect your suggestion. Additionally, we have update the body of the manuscript to reflect generalized anxiety disorder throughout.

We appreciate the reviewer’s suggestion to de-emphasize the association between regional insecurity and anxiety. As this was a core research question, we have retained this framing but revised the manuscript to clarify that the lack of association observed suggests the need for more nuanced or longitudinal research on this topic. To reflect this, we now place greater emphasis in the Discussion and Abstract on the protective effects of housing and vaccination, which yielded significant findings. This adjustment maintains the integrity of our research question while aligning with the reviewer’s suggestion to avoid over-interpreting a null association.

Some sections of the manuscript appear incomplete. I advise the authors to use the STROBE guidelines to complete all missing aspects, particularly in the Methods and Discussion sections.

Thank you for this suggestion, we have implemented changes throughout the manuscript to align with the STROBE guidelines.

Section-by-Section Specific Comments

Introduction
The research problem is clearly stated in paragraphs 2 and 3. However, what is missing is a description of the burden (epidemiological burden, economic burden, social burden, etc.) of anxiety disorders, supported by the evidence, to back up this stated problem. It is important to expand on the state of the art with statistical data.
We have added additional references and information pertaining to the current knowledge surrounding the burden of disease (lines 108-111). We do note that data surrounding GAD in our region and population of focus is limited, so we are only able to provide more broad data points.

The purpose could be a little clearer. Sometimes the authors state as follows: “This analysis explores the interplay of regional insecurity, environmental degradation, and the imminent threat of emerging infectious diseases, such as COVID-19 impacts anxiety levels” (Lines 90-92), sometimes as follows: “We aim to contribute to a nuanced understanding of the mental health challenges faced by the DRC population and offer insights, which could inform integrative policy interventions.” (Lines 92-94).

This is an excellent point and have further clarified the purpose within the background section in lines 103-105 and 133-140.

Methods
The Methods section contains very relevant information describing the research approach adopted. However, this information seems to be jumbled together, creating some confusion for the reader. To make this section much clearer for potential readers, I suggest subdividing it into the following sub-sections: study design; study setting; population and sampling; data collection methods and tools; data collection procedure; data analysis; data management and ethics, etc. Please use the STROBE guidelines to further clarify this section.

Thank you very much for this feedback, we have implemented the suggested STROBE guidelines and made the methods structured per your guidance. This has greatly improved readability, thank you!

The authors’ state: “phone interviews using a structured tablet-based questionnaire in SurveyCTO were conducted and typically took 15 to 30 minutes to complete. (Lines 106-107). Does this suggest that every participant in the study had a telephone? What then were all the inclusion and exclusion criteria for the study?
Thank you for this thoughtful question. Yes, all participants included in this analysis had access to a working telephone, though it was not required that they personally own the device. This clarification has been added to the manuscript on lines 155–157. We have also elaborated on the sampling methodology and added further detail on inclusion criteria to ensure transparency.

Apart from generalized anxiety disorders, which were measured using the 7-item generalized anxiety disorder scale (GAD-7), what other tools did the authors use to collect other data such as those mentioned on lines 110-111? If possible, it will be important to describe them very briefly as they have not been added as supplementary files.

Thank you for this comment. Apart from the GAD-7, all other variables used in this analysis were collected through individual categorical or ordinal survey items designed for the broader COVID-19 Vaccine Rumors Study. These items were not drawn from standardized psychological instruments, but were developed to capture relevant demographic information (e.g., age, gender, profession, housing type), COVID-19 perceptions (e.g., perceived severity, susceptibility, awareness), and protective behaviors (e.g., vaccination status). Given their simplicity, these items were not included as a supplementary file.

Results
In the following finding: “The full multivariate regression model indicated no significant association between insecure regions and mild to severe anxiety within this sample (aOR: 1.00, 95% CI: 0.70, 1.43)” (Lines 169-170), the authors note that there is no association between “insecure regions” and “anxiety disorder”, whereas this association is found elsewhere. Is this a problem of study design, information bias, measurement bias, etc.? It is important to discuss this result in depth and to call for further in-depth studies in this context.

We appreciate the reviewer’s concern and agree that significant associations between conflict and anxiety have been documented elsewhere. However, we believe the lack of a significant association in our analysis may reflect both context-specific resilience mechanisms and methodological limitations, which we already discuss in the manuscript (see Discussion, lines 370–391 and 435-460). We elaborate on the possible mismatch between the GAD-7 and cultural/linguistic frameworks within the DRC, as well as potential selection bias due to phone access. Additionally, we raise the possibility that HCWs in this setting—due to prior experiences with Ebola outbreaks—may demonstrate heightened preparedness and mental resilience. Given the complexity of mental health in conflict zones, we agree further longitudinal and qualitative research is warranted and have added language to emphasize this more clearly.

Normally, this result (aOR: 1.33, 95% CI: 0.99, 1.80) suggests an absence of association between gender and anxiety risk. Figure 3 shows this clearly. If you believe that there is indeed an association, it is necessary to round the lower limit of the confidence interval from 0.99 to 1.00. This would avoid passing through 1 in the confidence interval between the lower and upper limits. If you agree with me, please also amend the abstract.

We appreciate the reviewer’s observation. However, we respectfully disagree with the suggestion to round the lower limit of the confidence interval from 0.99 to 1.00. Rounding the CI in this manner would imply statistical significance where it does not exist based on the original regression output. To maintain accuracy and scientific rigor, we have retained the original confidence interval but clarified the language in the manuscript to emphasize that this finding is suggestive but not statistically significant.

The authors observed an increase in the prevalence of anxiety disorders at a given time: “We observed spikes in anxiety prevalence during the months of February and July” (Line 196). Did this correspond to a wave (outbreak) of COVID-19 cases, or is there another explanation? If possible, discuss this increase in the prevalence.

We thank the reviewer for this thoughtful comment. We agree that the observed spikes in February and July merit consideration. In the Results section, we acknowledge these patterns and in the Discussion offer potential contextual explanations (e.g., periods of unrest), while also noting limitations due to the cross-sectional nature of the data and unstratified province-level timing. As our dataset was not designed for time-series analysis, we have chosen to refrain from over-interpreting these fluctuations, but we agree that they may warrant further exploration in future studies. Additional discussion has been added in lines 439-443 to clarify these points.

Discussion
An anxiety disorder prevalence 10 times lower than the international literature, yet in the context of emerging COVID-19 and insecurity? This should be widely discussed.
Thank you for this important observation. We agree that this discrepancy is a critical and intriguing finding. We have discussed this extensively in the manuscript (lines 364–391 and lines 410–445), where we contrast our observed 4% prevalence with global estimates around 41%. We explore several possible explanations, including resilience among DRC healthcare workers due to prior experience with Ebola outbreaks, as well as multiple methodological considerations such as the use of the GAD-7 (which is not yet validated in the DRC), potential response and selection biases from phone-based survey methods, and the timing of data collection after the initial COVID-19 waves. We also acknowledge these limitations in the dedicated section toward the end of the Discussion. At this time, we believe this issue is well addressed in the manuscript, but we would be happy to further emphasize these points if the editor feels additional clarification is needed.

There are at least 6 types of anxiety disorder: panic disorder, agoraphobia, generalized anxiety disorder (GAD), social anxiety disorder, specific phobia, and separation anxiety disorder. In this study, the authors dealt with only one type: GAD. The Limitations of the study sub-section would mention the fact that the study was limited to this type of disorder and would justify why other types were not considered.

We appreciate the reviewer’s thoughtful comment regarding the scope of anxiety disorders addressed in our study. As correctly noted, the study focused exclusively on generalized anxiety disorder (GAD), assessed using the GAD-7 instrument. This limitation is due to the structure and intent of the parent study, which centered on COVID-19 vaccine misinformation and attitudes, not mental health as a primary outcome. The inclusion of GAD-7 was a supplemental effort initiated by the first author to explore mental health trends within available resources and time constraints.

Other types of anxiety disorders—such as panic disorder, social anxiety disorder, and specific phobias—were not assessed, as validated screening tools for those disorders were not part of the survey design, and the study budget and ethical approvals did not permit adding additional validated instruments at the time.

We have added clarification of this limitation in the Discussion section (under "Limitations"), noting that our findings are restricted to GAD and may not reflect the broader burden of anxiety disorders in the DRC. We also call for future research that employs a more comprehensive battery of mental health assessments to capture a wider range of anxiety and mood disorders within conflict-affected and under-resourced settings.

Reviewer #2: Thank you for giving me the opportunity to review this work. Please find a summary of my review
Section-wise Review & Suggested Edits
1. Title & Abstract
• The title should be concise and accurately reflect the core argument or findings of the paper. Consider making it more engaging.
• The abstract should provide a clearer summary of the research question, methodology key findings, and significance. If possible, make it structured.

Thank you for this valuable feedback. We have revised the title to more directly reflect the core findings of the study and improve reader engagement. Additionally, the abstract has been restructured to follow a clear, structured format, and updated to more clearly summarize the research question, methodology, key findings, and their broader implications.

2. Introduction
Strengths:
• The introduction provides background on the topic.
Areas for Improvement:
• The problem statement should be clearer. Why is this topic important?
• The research gap should be explicitly stated—how does this article contribute to the existing body of knowledge?
Suggested Edits:
• Strengthen the opening by clearly stating the research question or main argument.

Thank you for these insightful suggestions. In response, we have expanded the problem statement in lines 103–111 to better articulate the importance of understanding anxiety within the DRC context. We more explicitly identify the research gap and our contribution to the literature on mental health in conflict-affected, low-resource settings in lines 125–126. Additionally, we clarified the specific research question and objectives in lines 136–140 to ensure the focus of the study is clearly communicated to readers.

3. Literature Review / Background
Strengths:
• Covers relevant sources related to the topic.
Areas for Improvement:
• Some sections lack sufficient citations. Claims should be backed by evidence from existing studies.
• The connection between previous research and the current study should be clearer.
Suggested Edits:
• Add more references where needed.
• Clearly state how the reviewed literature informs the research.

Thank you for this thoughtful feedback. In response, we have added additional references in lines 108–111 to better support the contextual claims made regarding the burden of anxiety disorders and mental health challenges in conflict zones. We have also clarified how previous research informs the present study in lines 103–105 and 125–129, emphasizing the relevance of existing gaps in the literature and how this study contributes to addressing them.

4. Methodology (If Applicable)
Strengths:
• Describes the approach used for data collection or analysis.
Areas for Improvement:
• Lacks detail on why a particular methodology was chosen.
Suggested Edits:
• Provide a rationale for the chosen research method.
• Ensure reproducibility by adding sufficient methodological details.

Thank you for this valuable feedback. We have added further explanation regarding our rationale for selecting a cross-sectional phone-based survey design, including considerations around feasibility and context, in lines 144–160. Additionally, we restructured the Methods section for improved clarity and have expanded our methodological descriptions in lines 173–244 to better support reproducibility and transparency.

5. Results & Discussion
Strengths:
• The discussion touches on key findings.
Areas for Improvement:
• The results should be explicitly stated before discussion.
• The discussion should connect findings to existing literature—do they support or contradict previous studies?
• Consider structuring this section more clearly.
Suggested Edits:
• Clearly separate results from discussion for clarity.
• Use subheadings to organize findings logically.

Thank you for these helpful suggestions. The results are presented in a standalone section preceding the discussion, in line with standard reporting practices. To enhance clarity, we have added subheadings within the Results section to better organize and highlight key findings. We also expanded the Discussion section to more directly engage with relevant literature: lines 366–382 and 384–389 discuss how our findings diverge from existing research, while lines 393–396 and 410–411 emphasize areas of alignment. These additions aim to strengthen the connection between our findings and the broader body of literature.

6. Conclusion & Recommendations
Strengths:
• Summarizes key points.
Areas for Improvement:
• The conclusion should more explicitly state the study's contribution.
• Practical implications or future research directions could be discussed.
Suggested Edits:
• Reinforce the significance of the findings.
• Suggest future research directions to strengthen impact.

Thank you for this thoughtful feedback. We have revised the conclusion to more clearly articulate the study’s key contributions in lines 487–492, specifically emphasizing the relevance of our findings to mental health resilience research in conflict-affected settings. Additionally, we have expanded the section to better outline the practical implications of our work and suggest concrete directions for future research in lines 489–494. These edits aim to more strongly reinforce the significance and potential impact of the study.

7. Language & Formatting
Areas for Improvement:
• Some grammatical errors and obstinate phrasing should be revised.
• Ensure consistency in referencing style (APA, MLA, etc.).
Suggested Edits:
• Proofread for grammatical and syntactical errors.
• Use clear and precise academic language.

Thank you for this feedback. We have thoroughly reviewed and revised the manuscript for grammatical and syntactical accuracy, as well as overall clarity and readability. We also confirm that the reference style is consistent with PLOS journal guidelines, following the standard numbered format aligned with AMA/Vancouver citation style.
